# Changes in benthic community structure and sediment characteristics after natural recolonisation of the seagrass *Zostera muelleri*

**DOI:** 10.1038/s41598-018-31398-2

**Published:** 2018-09-05

**Authors:** Carolyn J. Lundquist, Tracey C. Jones, Samantha M. Parkes, Richard H. Bulmer

**Affiliations:** 1 National Institute of Water and Atmosphere (NIWA) Ltd, Auckland, New Zealand; 2 Institute of Marine Science, University of Auckland, Auckland, New Zealand

## Abstract

Macrofauna are important contributors to estuarine ecosystem services within and outside of seagrass beds. Here we documented the natural recolonisation of a temperate seagrass (*Zostera muelleri*) community over 15 years in an urban estuary (Waitemata Harbour, North Island, New Zealand). We also investigated the change in macrofaunal communities in relation to seagrass cover over time, from transition from bare sandflat to seagrass. Colonisation by seagrass was associated with an increase in macrofaunal species diversity (from an average of 32 species per core in 2001 to 46 species per core in 2015) and abundance (from 482 to 2273 individuals per core), as well as an increase in sediment mud (from 4.09% to 12.37%) and organic matter content (from 0.90% to 1.41%). The most abundant species within both seagrass and adjacent unvegetated sandflat were similar, the polychaetes *Heteromastus filiformis*, *Aricidea* sp., and *Prionospio aucklandica*, and the amphipod *Paracalliope novizealandiae*. The difference in macrofaunal community structure between seagrass and unvegetated sandflat was primarily associated with higher abundance of *P*. *novizealandiae* and lower abundance of *Pseudopolydora* sp. in seagrass. A successional effect was observed in macrofaunal communities over the 15 years following seagrass expansion, primarily associated with an increase in the abundance of *Aricidea* sp., *H*. *filiformis*, and *P*. *novizealandiae*, and a reduction in the abundance of the bivalve *Linucula hartvigiana*. This study is the first to document long-term changes in seagrass and their associated communities during a natural recolonisation event, providing insight into timeframes required both for the regrowth of a seagrass meadow from initial colonisation of individual patches, as well as the trajectories and timeframes of change from a sandflat to a seagrass-associated macrofaunal community. This research enhances our understanding of how changes in seagrass distributions due to seagrass loss or restoration may affect macrofaunal community composition and ultimately ecosystem function.

## Introduction

Seagrass beds are a major component of estuarine and coastal ecosystems, influencing a number of important ecosystem services, including coastal stabilisation, nutrient and carbon cycling and storage, and trophic transfer^1–3^. They play an important role structuring physical and biological aspects of intertidal areas, adding to the diversity in these highly productive ecosystems^4–6^. They provide important structural habitat for juvenile fish, although this role is likely limited in New Zealand as most seagrass is intertidal^4,7^. They modify the hydrodynamic environment, facilitating settlement of planktonic larvae and fine sediment^8–10^. They also affect the habitat available to infauna and epifauna, increasing the variety of microhabitats around leaves and root-rhizomes, and altering the predator-prey relationships (inhibiting foraging, sheltering from predation)^8,11,12^. In turn, macroinvertebrate faunal communities interact with and support many of the ecosystem services provided by seagrass beds^13–15^.

Despite the high value of ecosystem services provided by seagrass beds, major declines in seagrass beds have occurred, with an estimated 110 km^2^ yr^−1^ lost globally since 1980^16,17^. The main factors for this are thought to be eutrophication and increasing water column turbidity, which limit photosynthesis and can result in smothering of seagrass meadows^18^; these adverse environmental conditions are also associated with decreased resilience to disease^16^. In New Zealand *Zostera muelleri* has shown major decreases in area during the last century, particularly in highly impacted harbours such as Tauranga Harbour (34% decline in seagrass area from the 1940s through the late 1990s) and Porirua Harbour (50% decline from the 1960s through to the 1980s)^19,20^. Anecdotal records suggest that there were extensive seagrass meadows prior to European colonisation in Waitemata Harbour^21^, yet these had declined to covering only 60 ha by the 1990s^19^. However, a number of seagrass meadows in New Zealand are now rebounding, with increases in seagrass area observed in recent decades (for example, seagrass beds in Manukau, Whangarei, Waitemata Harbours)^22,23^. This increase appears to be largely unexplained, and uncoupled from any targeted management practices to improve seagrass abundance.

Seagrass in New Zealand is not as extensive or diverse in species number as those overseas, with only one species of seagrass occurring, *Z*. *muelleri* Irmisch ex Asch. (Zosteraceae)^24^. Studies of macrofaunal community structure in New Zealand estuaries show distinct differences in macroinvertebrate communities between locations with high *Z*. *muelleri* biomass and unvegetated sediment^25,26^. However, there have been no studies that look at what affect naturally re-occurring seagrass populations have on macrofaunal community assemblages over time (and the associated impact on ecosystem services). In addition, although macroinvertebrate associations with seagrass beds have been studied fairly extensively, most studies have been of short duration (1–2 years), limiting conclusions to seasonal differences^13,25–28^).

Rehabilitation and restoration of seagrass species are occurring globally, and restoring declines in seagrass meadows have been the subject of a number of restoration efforts in New Zealand^29,30^. These transplant/translocation efforts have varied in success, with one (Whangarei Harbour) cooccurring with natural recolonisation throughout the harbour that was too widespread to have been generated by the effect of the transplants alone^20,22^. In Manukau Harbour, unsuccessful efforts at transplanting seagrass were assumed to be due to hydrodynamic effects associated with storm events that dislodged seagrass plants^31^. Natural recolonisation of *Z*. *muelleri* has been detected in recent decades at a long-term estuary monitoring site within the Waitemata Harbour, with the area adjacent to the monitoring site containing few remnant patches of <1 ha in 1996 and expanding to >40 ha of mostly contiguous intertidal seagrass meadow in 2015. This natural recolonisation provides an opportunity to fill important gaps in our understanding of the response trajectory of benthic communities to seagrass recolonisation. This is particularly valuable given the interest in understanding the role of seagrass not just in providing habitat structure and supporting high species richness, but also in regard to other ecosystem services provided by seagrass-associated communities such as nutrient and carbon cycling and storage, and trophic transfer^1,32^.

The aim of this study was to determine whether changes in abundance and diversity of benthic communities and sediment characteristics occur following the recolonisation of seagrass populations. We hypothesise that (a) community assemblages and sediment characteristics are different at the beginning of this re-establishment period and fifteen years following re-establishment, and (b) sampling positions inclusive of more recent seagrass colonisation show differing community assemblages to older seagrass patches, i.e., there is a successional effect. This research will enhance our understanding of timeframes and trajectories of macrofaunal communities associated with natural recolonisation by seagrass meadows, and how changes in seagrass distributions due to seagrass loss or restoration may affect macrofaunal species richness, abundance, and community composition, ultimately resulting in changes in ecosystem function.

## Methods

### Study site

**Figure 1:**
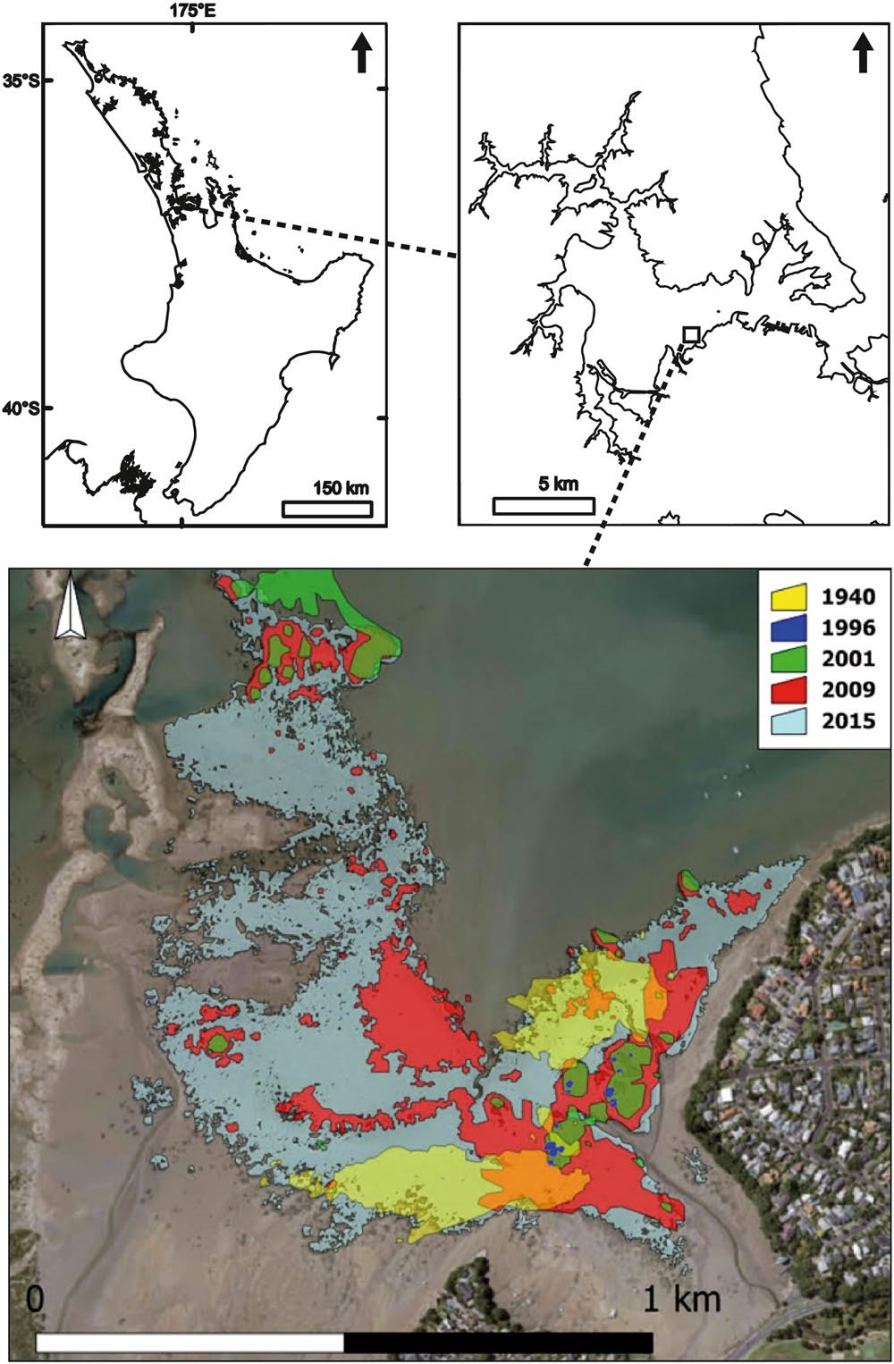
Top left and right: Location of study site. Bottom: Temporal changes in seagrass cover from 1940 to 2015 according to aerial photos (created using Manifold GIS version 8, satellite imagery sourced from Auckland Council under Creative Commons 4.0 licencing, https://data.linz.govt.nz/layer/88142-auckland-0075m-urban-aerial-photos-2015–16/).

Waitemata Harbour, North Island, New Zealand (Fig. 1) is a large, well-mixed estuary in urban Auckland, covering an area of ~80 km ^2,33^. The estuary is 36% intertidal, with a mean depth of 4.28 m, and the tidal range is 2.71 m during spring tides, 2.32 m during mean tides, and 1.94 m during neap tides^33^. While extensive seagrass beds dominated large areas in the Waitemata Harbour when Europeans arrived in New Zealand in the 18^th^ century^21^, seagrass meadows had declined by the 1931 to only small remnant patches, and were not observed at any of the five monitored sites when a long-term estuary monitoring programme was established by the regional authority (Auckland Council) in 2000^23^.

The study site (36° 50′ 45′′ S, 174° 43′ 02′′ E) is located on the intertidal flat on the eastern side of Te Tokoroa Reef near Meola and Motions Creek, with small patches of seagrass apparent in aerial photography in 1996 adjacent to the monitored site, which have since expanded to >40 ha in 2015. Seagrass aerial images (1940 to 2015) were obtained from Auckland Council aerial imagery databases under Creative Commons 4.0 licencing. The images were taken at low tide during calm water conditions so that seagrass beds were visible (Fig. 1). The images were then uploaded and georeferenced using Manifold GIS software version 8 (Manifold Net Ltd, Carson City, Nevada). The outline of all patches of seagrass were then carefully traced for each aerial image (Fig. 1).

As part of regional government monitoring of this harbour, regular sampling of sediment trapping, suspended sediment and sediment chemistry are also collected in aligned monitoring at the hard substrate Meola Reef within 500 m of the estuary monitoring site, briefly summarised here to provide background context on trends in water quality and sediment deposition associated with land-based sediment and nutrients, and the presence of other contaminants associated with ground water runoff. Sedimentation rates collected as part of a subtidal reef monitoring programme since 2001 show no evidence of a directional change, and are unlikely to have declined since 2001; rather, measures of sediment deposition and suspended sediment concentrations suggest an increase in sedimentation^34^. Water quality measurements indicate increasing trends in extractable metal content (Cu, PB, Zn) at the Motions Creek inner site (one of the primary freshwater creeks upstream of the seagrass bed) from 2004 to 2014, and decreases in PAHs, though PAH concentrations remain above threshold contaminant levels for the harbour^35^.

### Sampling protocol

The monitoring site was a 50 m × 180 m area, divided into 12 equal sections, with sampling occurring bimonthly from October 2000 to February 2010 and from August 2015 to December 2015, with an additional sampling event in March 2012. During each sampling event, one macrofaunal core (13 cm diameter, 15 cm deep) was collected from a random position within each of the twelve sectors to estimate macrofaunal abundance. Two surficial sediment cores were collected on each sampling occasion, avoiding seagrass biomass, one to determine grain-size and organic content and the other for sediment chlorophyll *a* analysis, with each being a composite of sediment cores (2 cm deep, 2 cm diameter) from six random locations within the site.

Ten additional transects were sampled in March 2012 to allow for examination of relationships amongst: sediment characteristics, position relative to shore, seagrass density and biomass, and macrofaunal community. Transects were separated by at least 10 m, and were placed perpendicular to the shore. Five positions were sampled at each transect: 10 m outside of the seaward seagrass boundary (SeawardSF); 10 m inside of the seaward seagrass boundary (SeawardSG); in the centre of the seagrass meadow (CentreSG); 10 m inside of the shoreward seagrass boundary (ShorewardSG); and 10 m outside of the shoreward seagrass boundary (ShorewardSF) (Fig. 1C). At each of the fifty transect sampling positions and twelve long-term monitoring positions (REEF), one macrofaunal core and two surface sediment cores were collected. Percent cover of sediment by seagrass was estimated at each position using a 0.5 m × 0.5 m quadrat. Length and width of ten seagrass blades were measured at each position.

Sample collection for this study was covered by Special Permit 597 issued by the New Zealand Ministry for Primary Industries to the National Institute of Water and Atmospheric Research Ltd.

### Sample Processing

Macrofaunal core samples were sieved on a 500 μm mesh, stained with Rose Bengal and preserved in 70% isopropyl alcohol. Macrofauna were identified and enumerated to the lowest practicable taxonomic level, usually to species. All vegetation was dried in a 60 degree C oven for four days, or until weights stabilised, and used to estimate above and below ground biomass of seagrass from each core.

Sediment cores were kept frozen in the dark prior to analysis. Sediment samples processed for grainsize analysis were homogenised, digested in hydrogen peroxide, wet sieved and dried at 60 °C, to separate percentage weights of gravel/shell hash (>2000 μm), coarse sand (500 to 2000 μm), medium sand (250 to 500 μm), fine sand (62.5 to 500 μm), and mud (<62.5 μm). Samples processed for organic content were dried at 60 °C for 48 h and then ashed for 5.5 h at 400 °C^36^. Chlorophyll *a* samples were freeze-dried, weighed and homogenised. Chlorophyll *a* was extracted from a subsample by boiling the sediment in 90% ethanol, and the extract was processed using a spectrophotometer. An acidification step was used to separate degradation products from chlorophyll *a*.

### Data Analysis

Analyses of macrofaunal data and their relationships to each other and environmental data were undertaken using the DIVERSE, MDS, SIMPER, BEST and PERMANOVA options of the PRIMER software version 6^37^. Total species, total individuals, and Shannon-Weiner diversity index were calculated using DIVERSE. Regression analysis was used to investigate linear trends for physical properties and macrofaunal data over time (AUTOREG procedure, SAS 9.3). Durban-Watson statistics were calculated to detect the presence of auto-correlation in the trend analysis. Where auto-correlation was indicated, increasing or decreasing trends were investigated by adjusting parameters and significance levels. Otherwise ordinary least squares regression was carried out. Regression analysis was linear unless a step trend was indicated or a logarithmic transformation was required. Trends in rank abundance of the top five species were calculated in Microsoft Excel (v. 2013).

Non-metric multidimensional scaling (MDS) of macrofaunal community data was used to assess temporal or positional variation in macrofaunal community composition. The data were raw, log and square root transformed to assess lowest stress, and square root transformation was subsequently used for all macrofaunal data. All ordinations were performed using Bray Curtis similarities. Similarity percentages of species contributions (SIMPER) using Bray Curtis similarity of macrofauna data was conducted to ascertain the main taxa contributing to the change in the communities.

To find the best match between macroinvertebrate sample patterns and the associated environmental variables such as a) Chlorophyll *a*, sediment grain size and organic matter, and b) to estimate the effect of duration of seagrass colonisation, seagrass % cover and seagrass % cover of the neighbourhood, data were analysed with Biota-Environment-Stepwise analysis (BEST) using Spearman’s ranked correlation method with D1 Euclidean distance resemblance. PERMANOVA was used to test for significant differences in environmental and macrofaunal data with position, with pairwise post-hoc tests used to identify differences (based on 2012 data).

## Results

### Seagrass recolonisation

Seagrass was observed in low coverage at the monitoring site prior to 2001, when small patches of seagrass were observed to colonise the bay. Since 2004 seagrass has increased exponentially from <1 to >40 hectares, with cover approximately doubling in size every two years (Fig. 1). Prior to 2009 seagrass cover was patchy, with this increasing from 2009 to 2015 to a high degree of continuous meadow, thus creating mosaics of different aged seagrass in a mostly continuous meadow (Fig. 1).

### Temporal trends

Trend analysis indicated that percent mud, abundance of individuals, and total seagrass area all increased significantly over the fifteen year period (p < 0.05). Organic content increased initially and then fluctuated from 2005 to 2015 at levels approximately double that observed when monitoring commenced. Total taxa also increased significantly over time (p < 0.05), with high inter-annual variability. Abundance of Chlorophyll *a* and Shannon-Wiener diversity did not show significant changes over that time (Table 1).

**Table 1:** Averages of environmental parameters, macrofaunal data, and total seagrass area over time.

Year	Mud %*	Chl a	Organic content*	Shannon-Wiener diversity	Total taxa*	Total individual*	Total seagrass area (ha)*
1940	—	—	—	—	—	—	7.29
1996	—	—	—	—	—	—	0.10
2000	4.09	7.28	0.90	2.06	32	482	—
2001	3.43	10.54	0.74	1.98	37	1016	3.56
2002	5.08	10.46	1.04	2.05	30	692	—
2003	6.74	6.42	1.08	2.51	47	1389	—
2004	6.47	5.36	1.20	2.29	38	1064	3.01
2005	7.61	18.45	1.64	2.29	46	1713	3.68
2006	8.05	7.80	1.73	2.65	45	939	—
2007	9.55	11.92	1.41	2.56	41	904	6.41
2008	8.36	6.42	1.48	2.43	47	999	—
2009	10.11	8.02	1.87	2.45	56	1623	13.65
2012	11.63	12.64	1.57	2.59	52	2125	31.56
2015	12.37	7.06	1.41	2.17	46	2273	43.51

Significant trends are marked with (*p < 0.05).

The dominance of taxa changed over time with the bivalve *Linucula hartvigiana* being the most dominant in 2000 and the polychaete *Aricidea* sp. being the most dominant in 2015. Declines in *L*. *hartvigiana* were observed harbour wide, suggesting this may be unrelated to seagrass recolonization^23^. The five most abundant species changed from the bivalves *Linucula hartvigiana*, the gastropod *Zeacumantus lutulentus*, and the polychaetes *Euchone* sp., *Macroclymenella stewartensis*, and *Aricidea* sp. in 2000, to the polychaetes *Aricidea* sp., *Heteromastus filiformis*, *Paracalliope novizealandiae*, *Boccardia syrtis*, and amphipods of the family Phoxocephalidae in 2015. Other contributors to top ranked abundance included the polychaete *Sphaerosyllis semiverricosa* (Supplementary Information Table 1).

**Figure 2:**
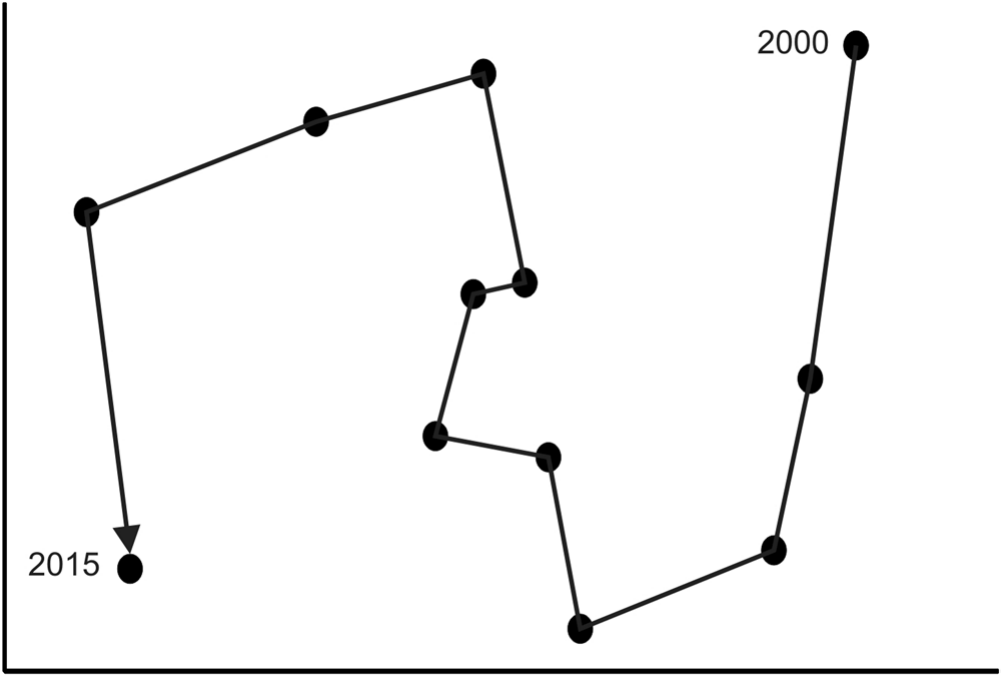
Square root transformed macrofaunal data showing the difference in community structure over time according to non-metric multi dimension space (nMDS). Stress level is 0.05.

Non-metric Multi-dimensional scaling (nMDS) showed clear differences with macrofaunal community structure changing over time (Fig. 2). SIMPER analysis indicated that between 2000 and 2015, the average dissimilarity was high (91.2%), driven primarily by changes in abundance of *Aricidea* sp. (29.2% contribution), *H*. *filiformis* (26.3% contribution), *L*. *hartvigiana* (9.3% contribution) and *P*. *novizealandiae* (8.4% contribution).

The relationship between environmental data and macrofauna was significant over time (p < 0.01 (Global BEST test (999 permutations)). The highest correlation with the macrofauna data was mud content (0.75 Rho) (Supplementary Information Table 2).

### Positional trends based on 2012 survey

Lower mud content was observed in the seaward and centre seagrass positions (SeawardSG, CentreSG) than other positions (p < 0.05). Chlorophyll *a* was higher in the seaward positions (SeawardSF, SeawardSG) than the other positions (p < 0.05). No significant difference in organic content was observed (p > 0.05). The total number of species was higher in the sandflat closest to the incoming tide (SeawardSF) than the shoreward and seaward seagrass positions (ShorewardSG, SeawardSG) (p < 0.05). The total number of species was also higher in the centre of the seagrass position (CentreSG) than the seaward seagrass position (SeawardSG) (p < 0.05). Total macrofaunal individuals was higher in the sandflat positions (SeawardSF and shorewardSF) than the seaward seagrass position (SeawardSG) (p < 0.05) (Table 2).

**Table 2:** Averages across transect samples of environmental parameters and macrofaunal data (2012) ±1 SEM.

Position	Mud %	Chl*a (ug/g)*	Organic content (%)	Shannon-Wiener Index	Total species	Total indivi-duals	Leaf length (mm)	Leaf width (mm)
SeawardSF	13.5 (1.58)	7.37 (0.60)	1.52 (0.13)	2.64 (0.04)	57 (0.83)	208 (21.13)	—	—
SeawardSG	6.85 (0.46)	7.11 (0.50)	1.56 (0.10)	2.36 (0.09)	39 (1.14)	126 (12.06)	144.55 (27.09)	2.51 (0.09)
CentreSG	9.86 (0.68)	9.05 (0.41)	1.49 (0.08)	2.43 (0.04)	54 (1.03)	166 (18.82)	181.44 (15.23)	3.36 (0.10)
ShorewardSG	11.28 (0.46)	9.62 (0.61)	1.78 (0.16)	2.38 (0.10)	47 (1.35)	151 (18.42)	164.55 (27.29)	2.93 (0.21)
ShorewardSF	13.84 (1.46)	8.54 (0.36)	1.64 (0.11)	2.56 (0.08)	47 (1.19)	177 (17.75)	—	—

**Figure 3:**
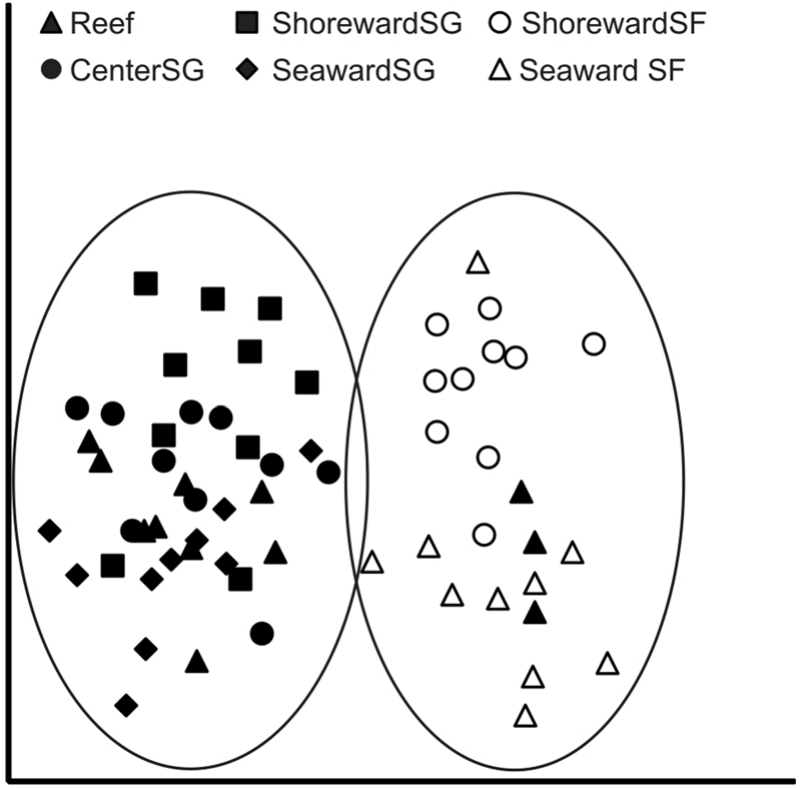
Square root transformed macrofaunal data showing the difference in community structure by position according to non-metric Multi Dimension Scaling (nMDS). Stress level is 0.18.

Macrofaunal taxa were strongly grouped according to position in the nMDS analysis (Fig. 3). Macrofauna in positions outside seagrass beds clump together, separate from macrofauna in seagrass positions which also clump together. There were three macrofaunal cores collected during annual monitoring (Reef) that clump with those collected from outside the seagrass meadow; these three cores occurred at the boundary of the seagrass meadow, with two cores having 0% seagrass cover and one with 15% seagrass cover (Fig. 3).

Despite the differences in community structure based on the nMDS, four of the top five most abundant species were identical across all positions, which were dominated numerically by the polychaetes *H*. *filiformis*, *Aricidia* sp., and *P*. *aucklandica*, and the amphipod *P*. *novizealandiae*. Other contributors to top ranked abundance between positions included *Nicon aestuarensis* and *Notoacmea scapha* (Supplementary Information Table 3). The difference in macrofaunal community structure between seagrass and unvegetated sandflat was primarily associated with higher abundance of *P*. *novizealandiae* and lower abundance of *Pseudopolydora* sp. in seagrass.

SIMPER analysis showed that higher abundance of *P*. *novizelandiae* in seagrass was the main contributor to differences in macrofaunal community structure between seagrass and sandflat positions (contributing 8.97% to 15.94% of differences), except for samples taken from outside the seagrass bed on the seaward side, in which lower abundance of the tube forming spionid polychaete *Pseudopolydora* sp. in seagrass was the largest contributor to the difference in community structures (14.22% to 16.44% contribution). There was no significant correlation with positional data to any environmental data (p > 0.7 (Global BEST test (999 permutations)).

Macrofaunal community composition in the positional analysis was strongly associated with all three seagrass cover variables (seagrass % cover, year of cover, and seagrass % cover of neighbourhood) (*Rho* 0.655, p < 0.001 (Global BEST test (999 permutations)) (Supplementary Information Table 4).

## Discussion

Our study investigated the impact of an expansion of an intertidal seagrass bed (from <1 ha in 1996 to >40 ha in 2015) on macrofaunal communities and sediment characteristics in a temperate estuary. Our results show that seagrass habitat has an overriding influence on macrofaunal community composition over space and time. To our knowledge, this is the first to investigate these long-term dynamics in the context of expansion of naturally re-occurring seagrass, with expansion associated with an increase in macrofaunal species abundance and diversity. As with seagrass, macrofaunal communities are known to play an important role delivering ecosystem services^13–15,32^. Our study indicates that seagrass meadows directly influence the species richness and community composition of macroinvertebrate communities, and that recovery of a natural seagrass meadow (i.e., without human intervention) can result in change in community composition from a sandflat-associated faunal community to one representing typical regional seagrass meadows within approximately a decade.

We observed a strong change in macrofaunal community structure over the 15 year study, from pre- to post-seagrass colonisation. This coincided with an increase in mud content at the study site. Seagrass canopies influence water current flow, dissipate turbulence and reduce wave action, reducing sediment resuspension and increasing sedimentation in seagrass beds^19,38^. Many of the species which contributed most strongly to the change in macrofaunal community structure are known to have a preference for higher mud content^39^, suggesting that the impact of seagrass on mud content may be a strong driver of macrofaunal change at this site. For example, the optimal mud content for *Aricidea* sp. and *P*. *novizealandiae* is 35–40%, while the optimal mud content for *H*. *filiformis* and *P*. *aucklandica* is 10–40% and 20–70% mud content, respectively^38^. We also observed an increase in the abundance of predator species (e.g., *P*. *novizealandiae* and oligochaetes), which is potentially an effect of dense seagrass, protecting and providing food for organisms within a wide range of functional groups, including deposit feeders, scavengers, grazers and predators, and thereby effecting population structure and productivity of key species^38^.

One New Zealand study found that seagrass beds appear to provide feeding and hiding grounds for organisms within a wide range of functional groups, including deposit feeders, scavengers, grazers and predators, which may help to explain the increase in total species over time observed in our study^25^. Changes in community composition could also be influenced by a cascade effect of seagrass detritus in the food web as macrofaunal structure is modified by detrital enrichment, in which seagrass is known to be important^40^. Detrital inputs also vary with sediment properties, i.e., mud compared to sand, with this affecting macrofaunal community response^41,42^.

Position relative to the shore and location of a seagrass bed relative to shore has been noted as important independent of size and complexity of the seagrass bed^26,28^. Changes in the composition of dominant macrofaunal species at varying positions in the seagrass bed from within the bed to outside the bed, and seaward and shoreward, also showed differences. A key difference between seagrass and unvegetated sandflat sites was the higher abundance of *P*. *novizelandiae*, and the lower abundance of *Pseudopolydora* sp. within seagrass. Interestingly at the CentreSG position the native limpet *Notoacmea scapha* ranked as a dominant species; this limpet ingests epiphytes that colonise seagrass blades. Although it is not restricted to the substrate of seagrass blades, in this substrate it has an ecophenotypic response with a smaller size and shell shape^43^.

Importantly we observed a high correlation between the presence and density of seagrass habitat and macrofaunal community structure (*Rho* 0.655). This direct comparison of macrofaunal species composition and abundance between seagrass habitat and unvegetated sandflats clearly indicated a difference in species composition between seagrass habitat and non-seagrass habitat. Hence although the wider Waitemata Harbour is also changing in mud content and dominant species^23^, we can be confident that the changes observed in this study are related to changes in seagrass habitat. Furthermore, the reduction in meadow fragmentation observed over the recolonisation process is likely to influence the ecosystem services provided by this seagrass bed; in New Zealand estuaries, patch size has been demonstrated to have a generally positive effect on diversity in seagrass and four other estuarine habitats (with the exception of mud habitats)^44^. Other studies have also demonstrated differences between shoreward and seaward patch edges, likely related to hydrodynamic disturbances^45^.

Given that we observed a strong relationship between seagrass cover and change in macrofaunal community structure over time, what we observed could be a move toward a ‘normal’ community associated with seagrasses, at least in this site, elucidating information on how community structure may evolve in a wider context. Differences in sampling methodologies (sieve mesh size, improved taxonomic resolution) complicate comparisons with other studies, however our measures of macrofaunal community structure within seagrass beds suggest that the study site supports a relatively high species diversity, including deposit feeders, scavengers, grazers and predators, which is consistent with elsewhere in New Zealand (typically around 20–25 species and 200 individuals per core)^25–28^, and higher than reported for *Zostera marina* beds elsewhere in the world (e.g., in the Baltic where ~5 species per core have been reported^13^).

In a review of the recovery of biological elements in intertidal areas, Borja, *et al*.^46^ found that it took >4 years for *Zostera noltii* and macroinvertebrates to recover from eutrophication pressure in a Portuguese estuary and between five and twenty years for vegetation/macroinvertebrates to recover after marsh and tidal restoration in Long Island Sound (USA). In Puget Sound, localised asynchronous declines and recovery of seagrass meadows occurred, whereas regional scale seagrass abundance appeared stable^47^. Large-scale seeding of seagrass meadows has resulted in rapid (within 10 years) recolonisation of 1600 ha, with high genetic diversity^48^.

## Conclusions

Macrofauna have a direct effect on ecosystem services through their influence on biogenic structure (i.e., burrows, trails, bioturbation) and nutrient fluxes and indirect effects such as predation, food webs, and detrital cycling. The influence of seagrass on macrofaunal communities seen here therefore highlights the importance of this marine flora on ecosystem service provision, as well as illustrating successful recovery of a seagrass-associated community after a natural recolonisation event. As our understanding of the relationship between seagrass and associated macrofaunal species and key ecosystem functions grow both in naturally recovering and artificially restored ecosystems^49^, this research will assist with predicting how changes in seagrass distributions due to seagrass loss or restoration may affect macrofaunal community composition and ultimately ecosystem function. While the contribution to ecosystem services through this medium is poorly understood, and potentially difficult to uncouple from existing contributions in an estuarine context, our study shows that this is an important consideration, particularly given the importance of biodiversity in reducing the risk of reaching non-reversible tipping points in both species diversity and ecosystem services^50,27^.

## Electronic supplementary material

Supplementary information
