# Supplementary material for: Changes in benthic community structure and sediment characteristics after natural recolonisation of the seagrass Zostera muelleri
Source: Sci Rep. 2018 Sep 5;8:13250. doi: 10.1038/s41598-018-31398-2 (PMC6125579; doi:10.1038/s41598-018-31398-2)
Supplement: Supplementary file 1 — Supplementary information [file 41598_2018_31398_MOESM1_ESM.docx]

**“Changes in benthic community structure and sediment characteristics after natural recolonisation of the seagrass *Zostera muelleri*”**

**Authors**

Carolyn J Lundquist*^1&2^, Tracey C Jones^1^, Samantha M Parkes^1^, Richard H Bulmer^1^.

^1^National Institute of Water and Atmosphere (NIWA) Ltd.

^2^Institute of Marine Science, University of Auckland.

*Corresponding Author: Carolyn.Lundquist@niwa.co.nz

**Supplementary Information**

Table 1: Rank dominance of top 5 macrofaunal species for all years at Meola Reef

| Year | *1^st^* | *2nd* | *3rd* | *4^th^* | *5th* |
| --- | --- | --- | --- | --- | --- |
| 2000 | *Linucula* | *Euchone* | *Aricidea* | *Zeacumantus* | *Macroclymenella* |
| 2001 | *Linucula* | *Euchone* | *Aricidea* | *Sphaerosyllis* | Oligochaeta |
| 2002 | *Linucula* | *Euchone* | *Aricidea* | *Sphaerosyllis* | *Prionospio* |
| 2003 | *Euchone* | *Austrovenus* | *Linucula* | *Sphaerosyllis* | *Heteromastus* |
| 2004 | *Euchone* | *Sphaerosyllis* | *Heteromastus* | *Aricidea* | *Prionospio* |
| 2005 | *Heteromastus* | *Euchone* | *Sphaerosyllis* | *Boccardia* | *Aricidea* |
| 2006 | *Heteromastus* | *Euchone* | *Sphaerosyllis* | *Aricidea* | *Macroclymenella* |
| 2007 | *Heteromastus* | *Sphaerosyllis* | *Euchone* | *Aricidea* | *Prionospio* |
| 2008 | *Sphaerosyllis* | *Heteromastus* | *Euchone* | *Pseudopolydora* spT | *Boccardia* |
| 2009 | *Heteromastus* | *Boccardia* | *Sphaerosyllis* | *Aricidea* | *Pseudopolydora* spT |
| 2012 | *Heteromastus* | *Aricidea* | *Paracalliope* | *Prionospio* | *Pseudopolydora* spT |
| 2015 | *Aricidea* | *Heteromastus* | *Paracalliope* | *Boccardia* | *Phoxocephalidae* |

Table 2: Associations between sediment characteristics and the macrofaunal community from 2000 to 2015. BIOENV (BEST, Primer v.6).

Variables include: 1 % Organics, 2 Chl*a*, ug/g 3 %fines, 4 %mud, 5 %>250µm. Grain sizes are as in methods section.

| No. of Variables | Spearman Rank Correlation (*Rho*) | Variables |
| --- | --- | --- |
| 1 | 0.750 | 4 |
| 2 | 0.665 | 3,4 |
| 3 | 0.562 | 2-4 |
| 3 | 0.542 | 3-5 |
| 4 | 0.483 | 2-5 |
| 3 | 0.468 | 1, 3-4 |
| 2 | 0.462 | 4-5 |
| 2 | 0.451 | 2,4 |
| 1 | 0.422 | 3 |
| 2 | 0.410 | 3,5 |

Table 3: Rank dominance table for Meola reef and transects 2012. Also includes 2012 annual sampling data (Reef).

| Position | 1st | 2^nd^ | 3^rd^ | 4th | 5^th^ |
| --- | --- | --- | --- | --- | --- |
| All data | *Heteromastus* | *Aricidea* | *Prionospio* | *Paracalliope* | *Boccardia* |
| Reef | *Heteromastus* | *Aricidea* | *Paracalliope* | *Prionospio* | *Pseudopolydora* |
| ShorewardSF | *Heteromastus* | *Pseudopolydora* | *Boccardia* | *Aricidea* | *Euchone* |
| ShorewardSG | *Heteromastus* | *Aricidea* | *Paracalliope* | *Prionospio* | *Boccardia* |
| CentreSG | *Heteromastus* | *Paracalliope* | *Prionospio* | *Aricidea* | *Notoacmea* |
| SeawardSG | *Heteromastus* | *Paracalliope* | *Prionospio* | *Aricidea* | *Boccardia* |
| SeawardSF | *Heteromastus* | *Prionospio* | *Boccardia* | *Nicon* | *Aricidea* |

Table 4: Associations between seagrass variables and sediment characteristics and the macrofaunal community. BIOENV (BEST, Primer v.6) based on 2012 data.

Variables are: 1 % Cover, 2 First cover, 3 Neighbourhood % cover, 4 % >250 µm, 5 % Fine Sand, 6 % Mud, 7 % Organics, 8 Chl*a*.

| No. of Variables | Spearman Rank Correlation (*Rho*) | Variables |
| --- | --- | --- |
| 3 | 0.655 | 1-3 |
| 2 | 0.650 | 1,3 |
| 2 | 0.636 | 2,3 |
| 1 | 0.624 | 1 |
| 2 | 0.614 | 1,2 |
| 4 | 0.604 | 1-3,7 |
| 4 | 0.591 | 1-3,5 |
| 1 | 0.591 | 2 |
| 4 | 0.591 | 1-4 |
| 4 | 0.589 | 1-3,6 |
